# The effect of education on the knowledge of patients with celiac disease 

**Published:** 2017

**Authors:** Farnoush Barzegar, Mohammad Rostami-Nejad, Hamid Mohaghegh Shalmani, Amir Sadeghi, Maryam Allahverdi Khani, David aldulaimi

**Affiliations:** 1 *Student Research Committee, Gastroenterology and Liver Diseases Research Center, Shahid Beheshti University of Medical Sciences, Tehran, Iran*; 2 *Gastroenterology and Liver Diseases Research Center, Research Institute for Gastroenterology and Liver Diseases, Shahid Beheshti University of Medical Sciences, Tehran, Iran.*; 3 *Basic and Molecular Epidemiology of Gastrointestinal Disorders Research Center, Research Institute for Gastroenterology and Liver Diseases, Shahid Beheshti University of Medical Sciences, Tehran, Iran*; 4 *Foodborne and waterborne diseases research center, Research institute for gastroenterology and liver diseases, Shahid Beheshti University of Medical Sciences*; 5 *Faculty of Medicine, Najafabad Branch, Islamic Azad University, Najafabad, Iran*; 6 *Department of Gastroenterology, South Warwickshire Foundation Trust, UK*

**Keywords:** Celiac disease, Patients Education, Knowledge

## Abstract

**Aim:**

The aim of this study was to investigate the effects of education on patients’ knowledge of celiac disease, in an Iranian population.

**Background:**

Education can increase patients’ knowledge regarding their disease, leading to improvements in their health.

**Methods:**

This cross-sectional study was conducted on patients who had been diagnosed with celiac disease. The patients attended an educational meeting in September, 2016. During the educational meeting information regarding the epidemiology, diagnosis and treatment of celiac disease was provided to the study subjects. Each patient completed a questionnaire regarding celiac disease before and after the educational meeting. The questionnaires were scored. Study data was analyzed using SPSS version 20.

**Results:**

90 patients were recruited (69 [77%] were women). Analysis of questionnaire responses showed that except for awareness of cross contamination with gluten, the education meeting significantly increased the knowledge of patients with celiac disease regarding epidemiology, diagnosis and treatment (p=0.001).

**Conclusion:**

The result of this study shows that an educational meeting can increase the knowledge of CD patients in treatment. Increasing patients’ knowledge may lead to improvements in patients’ health.

## Introduction

 Methods for improving patients’ knowledge regarding a disease, include patient-specific educational events (1). Educational events, along with other educational resources, can lead to changes in patients’ behavior and attitudes, helping them to overcome disease related problems (2). Traditional patient education programs will not have desired effects, without having a logical and systematic approach to changing patients’ behavior (3). Through patient education programs, individuals and groups can become empowered to change their own behavior and attitudes, and those of their local community. Understanding and knowledge of unknowns has made training an increasingly important issue (4). 

There are many diseases that impose significant burden on healthcare resources, among people in the community. At the same time, as addressing patient educational needs, a healthcare system must maintain the general health of the entire community: providing the necessary health services to all people. Through educational programs that increase knowledge and provide training, patients and their families can learn to improve their own well-being. 

Celiac disease (CD) is a lifelong condition that affects the small intestine. Symptoms can include diarrhea, bloating, weight loss and fatigue. It is diagnosed using common serology screening tests and taking small intestinal biopsies. Treatment is a lifelong gluten free diet (GFD). A GFD will often improve patients’ symptoms.(5) Patients with CD frequently complain about their limited knowledge regarding the diagnosis and treatment of their condition. Studies in the Middle East have shown that the prevalence of CD in Iran is about one percent (6). Lack of support for patients who have been recently diagnosed with CD may lead to, intentional or unintentional failure to comply with a GFD. Poor compliance with a GFD can have adverse health outcomes including ongoing symptoms and the development of complications such as an enteropathy-associated T-cell lymphoma (EATL) (7). Furthermore, symptomatic patients with CD may remain undiagnosed due to lack of healthcare awareness of the condition, despite the availability of sensitive and specific diagnostic tests. 

The purpose of this study was to evaluate the effect of a patient education meeting for patients with celiac disease on their knowledge about the diagnosis and treatment of CD. 

## Methods

This study was a cross-sectional study with the use of test-retest method. Ninety people, who had been diagnosed with CD, were included in a training program specifically designed to assess their awareness. The training program was held in the Research Institute of Gastroenterology and Liver Diseases, Shahid Beheshti University of Medical Sciences, Tehran in September, 2016. Criteria for entering the study included: definitive diagnosis of CD by gastroenterologists through serologic tests (tTG, IgA) and small intestinal sampling (according to Marsh’s classification) as well as the individual’s willingness to participate in the study. Exclusion criteria included a diagnosis of CD that had not been confirmed, age less than 15 years old and an incomplete questionnaire before or after the training program.

The instrument used in this study was a researcher-made questionnaire. Its reliability was confirmed by Cronbach's alpha analysis and its validity was confirmed by gastroenterologists. In the first part of the questionnaire, demographic characteristics of the patients were compiled, including: age, sex, educational level and location of the residence. The second part of the questionnaire consisted of 11 awareness-raising questions regarding CD: 2 questions were related to the epidemiology of the disease, 4 were related to the diagnosis, and the remaining questions were devoted to treatment and a GFD. In order to evaluate the performance objectives, in addition to using images and the statement of goals and questions, the learner was given the chance to achieve the desired goal. Questions were asked to encourage participant information processing and goal measurement. The contents of the training program were based on the most up-to-date and most reliable training guidelines in the world, based on Rothwell’s training design principles for celiac disease and the use of pilot test results. The training was done directly in two 4-hour lecture sessions, with question and answer sessions. 

Exploratory strategies including the use of images, statement of goals and questions related to each section before the beginning of the lecture, as well as training pamphlets and direct participation of patients in the training was used to attract patients. 

The presentation method of training contents was in accordance with the principles of instruction based on deductive, known to unknown, and generic to specific sorting methods. Contents relating to CD included definitions, epidemiology of the disease, diagnosis and treatment, including the GFD. Information regarding the GFD included examples of gluten-containing products, suspected gluten-free products and gluten-free products.

Prior to receiving the educational program, subjects completed a questionnaire. After completion of the educational program, a second questionnaire was completed. After collecting all open-ended questionnaires from patients, data was analyzed using SPSS 20 software. In order to classify the data, descriptive analysis was used and inference tests, including t test, one way anova and chi square test were used to find the relationship between two variables. Logistic regression model was also used to find the correlation between variables. P value less than 0.05 was considered for statistical significance. The study was approved by the medical ethics committee of Research Institute of Gastroenterology and Liver Diseases, Shahid Beheshti University of Medical Sciences, Tehran according to the Helsinki declaration (1395/47804). 

## Results

In this study 90 subjects were recruited. All the subjects had been diagnosed with CD and had been treated with a GFD for at least one year. Of these, 21 were males (23.3%) and 69 were females (76.7%). The mean age of patients was 47± 6.24 years old. 40% of the subjects in this study had university education and lived in Tehran. The demographic data of the surveyed individuals is presented in Table 1. 

**Table1:** Demographic information of investigated cases

		Number	Percent
Age	<<40	30	33.3%
	>40	60	66.7%
Gender	Female	69	76.7%
	Male	21	23.3%
living area	Tehran	36	40%
	Other cities	54	60%
Education	Under Diploma	26	28.9%
	Diploma	20	22.2%
	Academic	44	39.9%
Total		90	100

**Table2:** The status of answering questions of the questionnaire

	Mean		Standard deviation		P-Value
	Test	Retest	Test	Retest	
Question1	1.22	6.0	3.29	4.92	0.0001
Question2	6.16	6.07	2.88	1.73	0.0001
Question3	8.44	9.44	3.56	2.30	0.039
Question4	6.61	8.56	4.54	3.28	0.001
Question5	8.17	9.56	2.59	1.37	0.0001
Question6	8.86	9.13	2.64	2.71	0.366
Question7	7.89	9.29	3.89	2.51	0.005
Question8	4.94	8.11	3.63	3.23	0.0001
Question9	4.22	8.33	3.42	2.99	0.0001
Question10	6.06	9.17	4.46	2.62	0.0001
Question11	5.96	8.83	2.87	2.25	0.0001

The results showed that there is no relationship between the level of training, the place of residence and sex with the level of awareness about CD (P> 0.05).

Questions of the questionnaire were divided into three categories: treatment, epidemiology and diagnosis. The mean scores before training were 7.16, 2.72 and 6.81 respectively, while the average after the training changed to 8.98, 7.16 and 9.06.

For inferential statistics, Wilcoxon test was used for intra-group comparison. The results showed that the mean level of awareness of patients in terms of epidemiology, diagnosis and treatment was significantly increased after the training (P=0.0001). However, regarding the probability of contamination with gluten, the results showed no significant correlation with training level (P> 0.05) (Table 2).

## Discussion

CD is a chronic gastrointestinal disorder that develops in genetically susceptible individuals and is treated with a GFD(8). Complications of CD include malabsorption (9). The treatment of CD includes lifelong patient adherence to a GFD. Delayed diagnosis and failure to educate patients with CD can lead to ongoing symptoms and the complications. Patient education can improve and enhance knowledge and skills in different fields.(10) Through education programs it is possible to enhance the capabilities of patients and their families (11). Education techniques include the use of training design patterns - tools for teaching and training. (12) Application of theories such as the knowledge of unknowns and research to find new techniques and tools to solve community problems, can help patients and their communities improve their knowledge, in developed and developing countries (13).

The effect of training on treatment has already been considered and studied in diabetes, chemotherapy etc. Increasing knowledge, understanding and application of knowledge by patients and their communities should be accompanied by healthcare benefits.

This study showed that an education program for patients led to an increase in the level of knowledge and understanding. This finding is similar to the results of previous research on the effect of training on treatment (14, 15). After controlling for the effect of awareness, the effect of the patients' gender on self-care was not significant. Our results are similar to the results of the training study on treatment reported by Baghaei et al and Hatamloo.(16, 17)

In a study by Zippser et al, in the United States, it was reported that CD was diagnosed in 11% of patients by family physicians and internists and in 65% of patients by gastrointestinal specialists. The study concluded that physicians lacked awareness of CD, at the time of onset of the symptoms. The study also validated the use of serological tests (18). 

Assiri and colleagues in Saudi Arabia, conducted a cross-sectional descriptive study, investigating physicians' awareness of CD. The survey of medical staff was performed in primary care, intermediate care, and tertiary level care in public and private hospitals within Riyadh. The results of this study showed that awareness level among physicians - including counselors - about the diagnosis of CD is low. Improved education regarding CD is therefore required for medical staff. (19). 

This study has some limitations. Only patients who participated in the educational meeting were assessed regarding their knowledge of CD and medical staff were not assessed. Therefore, to increasing the generality of findings, performing similar studies with a larger sample size is recommended. 

Despite these limitations, it can be concluded from this study that an educational program for patients with CD has a significant effect on increasing knowledge regarding the diagnosis and treatment of CD.
